# Effect of salts on the Co-fermentation of glucose and xylose by a genetically engineered strain of *Saccharomyces cerevisiae*

**DOI:** 10.1186/1754-6834-6-83

**Published:** 2013-05-29

**Authors:** Elizabeth Casey, Nathan S Mosier, Jiri Adamec, Zachary Stockdale, Nancy Ho, Miroslav Sedlak

**Affiliations:** 1 Laboratory of Renewable Resources Engineering, Purdue University, West Lafayette, IN 47907, USA; 2 Department of Chemical Engineering, Purdue University, West Lafayette, IN 47907, USA; 3 Department of Biochemistry, University of Nebraska, Lincoln, NE 68588, USA; 4 Department of Chemistry, University of Illinois, Champaign, IL 61820, USA; 5 Department of Agricultural and Biological Engineering, Purdue University, West Lafayette, IN 47907, USA

**Keywords:** Yeast (*S. cerevisiae*), Xylose, Inhibition, Salt, Ethanol, Fermentation

## Abstract

**Background:**

A challenge currently facing the cellulosic biofuel industry is the efficient fermentation of both C5 and C6 sugars in the presence of inhibitors. To overcome this challenge, microorganisms that are capable of mixed-sugar fermentation need to be further developed for increased inhibitor tolerance. However, this requires an understanding of the physiological impact of inhibitors on the microorganism. This paper investigates the effect of salts on *Saccharomyces cerevisiae* 424A(LNH-ST), a yeast strain capable of effectively co-fermenting glucose and xylose.

**Results:**

In this study, we show that salts can be significant inhibitors of *S. cerevisiae*. All 6 pairs of anions (chloride and sulfate) and cations (sodium, potassium, and ammonium) tested resulted in reduced cell growth rate, glucose consumption rate, and ethanol production rate. In addition, the data showed that the xylose consumption is more strongly affected by salts than glucose consumption at all concentrations. At a NaCl concentration of 0.5M, the xylose consumption rate was reduced by 64.5% compared to the control. A metabolomics study found a shift in metabolism to increased glycerol production during xylose fermentation when salt was present, which was confirmed by an increase in extracellular glycerol titers by 4 fold. There were significant differences between the different cations. The salts with potassium cations were the least inhibitory. Surprisingly, although salts of sulfate produced twice the concentration of cations as compared to salts of chloride, the degree of inhibition was the same with one exception. Potassium salts of sulfate were less inhibitory than potassium paired with chloride, suggesting that chloride is more inhibitory than sulfate.

**Conclusions:**

When developing microorganisms and processes for cellulosic ethanol production, it is important to consider salt concentrations as it has a significant negative impact on yeast performance, especially with regards to xylose fermentation.

## Background

A number of technical hurdles could hinder the commercialization of cellulosic biofuels [1]. One major challenge is the engineering of robust, process-relevant, industrial microbes that are capable of mixed-sugar (hexose and pentose) fermentation with tolerance to inhibitors [2]. Significant progress has been made on the development of organisms capable of mixed-sugar fermentation (see [3] for a review). Metabolic engineering of *S. cerevisiae* has resulted in strains that can effectively utilize xylose in addition to glucose [4-9]. However, limited progress has been made on the development of industrial strains of multiple-sugar-fermenting microorganisms that are tolerant of harsh industrial conditions related to the conversion of lignocellulosic biomass to ethanol. Rather, most of the research focus has been on detoxification of lignocellulosic hydrolysates (see [10] for a review). Some detoxification processes may not provide the most economically feasible solutions while other may introduce potential inhibitors such as salt, so focus must return to developing organisms that can tolerate expected levels of the major inhibitors. This requires an understanding of how these inhibitors impact yeast fermentation performance, especially with regard to the fermentation of xylose. Xylose can represent as much as 50% of the available carbohydrates in lignocellulosic biomass.

The most commonly studied inhibitors found in lignocellulosic hydrolysates are weak acids, furan derivatives, and phenolic compounds [11-13]. However, salts, ionic compounds composed of cations and anions, must also be considered. Salts can originate from both the biomass itself [12] and from chemicals added during the processing of the biomass into fermentable sugars (e.g. for adjusting the pH of the enzyme hydrolysis and fermentation, and detoxification [10]) as well as during fermentation of the sugars to ethanol. For example, sulfuric acid, calcium hydroxide, and ammonia can be used as catalysts for pretreatment or process stream conditioning [14], and potassium hydroxide, sodium hydroxide and hydrochloric acid can be used for pH adjustment before and during fermentation [15]. Therefore, cations that could be expected in fermentation media include Na^+^, NH_4_^+^, and K^+^. Associated anions include Cl^-^ and SO_4_^-2^. A number of these ions have been shown to have a significant inhibitory effect on microorganisms that are considered for biofuel production. Specifically, they were found to reduce cell growth, sugar utilization rates, and ethanol productivity rates, while increasing ethanol yields and fermentation byproducts such as glycerol [16-18]. Two different inhibition mechanisms likely explain these results. When exposed to high salt concentrations, organisms can experience both osmotic stress and ion toxicity [19]. However, these previous studies have one major limitation: they were conducted with microorganisms capable of fermenting glucose, but not xylose. Therefore, the impact of these salts on xylose fermentation is unknown. Even in the absence of inhibitors, xylose fermentation by genetically engineered *S. cerevisiae* is slower than that of glucose fermentation [4,6,8]. The presence of inhibitors has been shown to exaggerate the difference between glucose and xylose fermentation rates [20-23]. Salt ions may have a similar, enhanced inhibitory effect on xylose fermentation and contribute to a significantly slower fermentation rate of lignocelullosic hydrolysates when compared to simple sugar cane or corn starch hydrolysates.

The goal of this study was to determine the effect of a variety of salt ions on the co-fermentation of glucose and xylose by using our engineered strain, *S. cerevisiae*, 424A(LNH-ST). We report the effect of different salt ions and concentrations on glucose and xylose consumption rates, ethanol production rates, and cell growth. Furthermore, we explored the effect of sodium chloride on xylose fermentation through a comprehensive analysis of intracellular metabolites involved in glycolysis and the pentose phosphate pathway.

## Results and discussion

### Impact on glucose and xylose consumption rates

**Figure 1:**
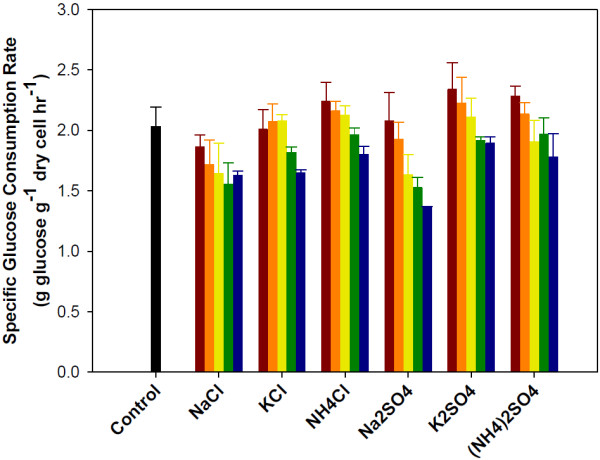
**Specific glucose consumption rates observed during the co-fermentation of glucose and xylose by *S. cerevisiae *with the addition of salts.** Salts concentration: no salt addition black bar, 0.1M red bar, 0.2M orange bar, 0.3M yellow bar, 0.4M green bar and 0.5M blue bar. Error bars represent standard errors. Experiments were carried out in duplicate.

**Figure 2:**
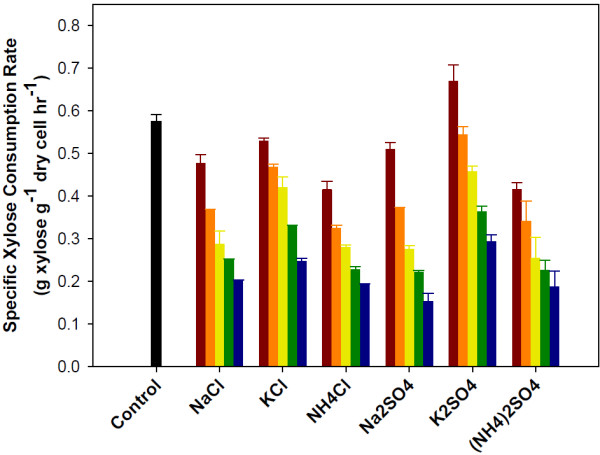
**Specific xylose consumption rates observed during the co-fermentation of glucose and xylose by *S. cerevisiae *with the addition of salts.** Salts concentration: no salt addition black bar, 0.1M red bar, 0.2M orange bar, 0.3M yellow bar, 0.4M green bar and 0.5M blue bar. Error bars represent standard errors. Experiments were carried out in duplicate.

To explore the effect of salts on glucose and xylose consumption, the specific consumption rates (for both glucose and xylose) were calculated for each fermentation condition using the model described in Methods. The results are summarized in Figures 1 and 2. No specific trend is observed between salt concentration and glucose consumption rates (Figure 1). At low salt concentrations (up to 0.2M), the only salt found to have any inhibitory effect on glucose consumption rates was sodium chloride, although the inhibitory effect was statistically insignificant. The remaining ion pairs (KCl, NH_4_Cl, Na_2_SO_4_, K_2_SO_4_, and (NH_4_)_2_SO_4_) appeared to have no or a slightly positive impact on glucose consumption at concentrations at or below 0.2M, increasing glucose consumption rates by up to 15% as compared to the control (Figure 1). Improved glucose utilization with salts has been reported previously with *Zymomonas mobilis *[24]. The presence of salts in fermentation media can lead to osmotic stress. *Saccharomyces* yeasts have several different mechanisms to combat osmotic stress, many of which require energy or carbon [25]. This need for additional energy and carbon could explain the enhanced glucose consumption rates at low salt concentrations. However, at higher salt concentrations, glucose consumption rates were decreased (Figure 1). At the most severe concentration tested (0.5M for cations paired with chloride and 1.0M for cations paired with sulfate), glucose consumption rate decreased between 12% (K_2_SO_4_) and 33% (Na_2_SO_4_) as compared to the control. However, salts might have greater inhibitory effects on other recombinant *Saccharomyces* yeast developed for glucose /xylose co-fermentation than the 424A(LNH-ST) strain, especially if the parent strain used for development was a laboratory strain derived from CEN.PK [26]. The parent strain used for the development of 424A(LNH-ST) is a robust industrial strain for ethanol production.

The effect of salts on xylose consumption was more severe than that for glucose consumption. A strong negative linear relationship was observed between xylose consumption rates and increasing salt concentration (Figure 2). Regardless of concentration, the presence of salt inhibited xylose consumption (the only exception is potassium sulfate at 0.1M). On average, xylose consumption rates were reduced by more than 60% compared to the control when salts were present at a concentration of 0.5M (0.5M for cations paired with chloride and 1.0M for cations paired with sulfate). Comparing the different salts, there is no significant difference in inhibition between the two anions (chloride vs. sulfate) despite of fact that sulfate salts in solution will yield twice the concentration of cations (Na^+^, K^+^, NH_4_^+^). This suggests that the anion also affects fermentation, with chloride being more inhibitory than sulfate. When comparing cations, significant differences exist between the three cations. Potassium containing salts are less inhibitory than the salts with sodium or ammonium (Figure 2). The impact of potassium and sodium ions on yeast has been widely studied [27]. Sodium ions are toxic to yeast. Under normal growth conditions yeast maintain a low intracellular sodium concentration. However, potassium is needed for many different physiological functions. Therefore yeast maintain a high intracellular potassium content. This may help explain why the impact of potassium salts on the fermentation performance of yeast was less severe than the other salts tested.

The results also show the difference in magnitude between glucose and xylose consumption. Looking at the results for the control, the specific glucose consumption rate is 2.05 g glucose g^-1^ dry cells hr^-1^ while the specific xylose consumption rate is only 0.57 g xylose g^-1^ dry cells hr^-1^. The xylose consumption rate is only about a quarter of the glucose consumption rate. This difference partially explains the reason why xylose fermentation is more affected by salt than glucose fermentation. A reduced consumption rate results in a reduced ATP generation rate. When combined with a reduced ATP yield (1.67 mol ATP mol^-1^ xylose compared with 2.0 mol ATP mol^-1^ glucose), the estimated ATP generation rate when xylose is the sole carbon source is approximately 20% of the ATP generation rate when glucose is fermented. Without an adequate supply of ATP, the yeast may have difficulties effectively mitigating the effects of osmotic stress and ion toxicity, resulting in increased salt inhibition when xylose is the primary carbon source.

To further explore the effect of salt on xylose fermentation, a series of fermentations of xylose (no glucose) with sodium chloride was conducted. The average xylose consumption rate results from two independent experiments are presented in Table 1. For comparison, the rates for glucose/xylose co-fermentation with sodium chloride are included. No significant difference between glucose/xylose and xylose only fermentations was determined using a student’s *t*-test (p = 0.05). Therefore, the effect of salt on xylose consumption under these experimental conditions is independent of the glucose utilization and the subsequent production of ethanol during the initial stage of fermentation (in this yeast strain, significant xylose consumption does not begin until glucose concentration falls below 10 g/l).

**Table 1:** Effect of sodium chloride salt on the consumption rates for xylose following glucose fermentation and xylose only fermentation

	**Specific xylose consumption rate (g xylose g**^**-1 **^**dry cells hr**^**-1**^**)**				
	**Glucose/xylose co-fermentation**		**Xylose only fermentation**		
**NaCl Conc. (M)**	***Average***	***Standard deviation***	***Average***	***Standard deviation***	***p-value***
0.0	0.526	0.091	0.575	0.022	0.651
0.1	0.482	0.014	0.476	0.029	0.698
0.2	0.379	0.046	0.366	0.003	0.764
0.3	0.287	0.020	0.287	0.044	0.994
0.4	0.225	0.013	0.250	0.003	0.266
0.5	0.141	0.008	0.204	0.000	0.058

### Impact on ethanol production

**Figure 3:**
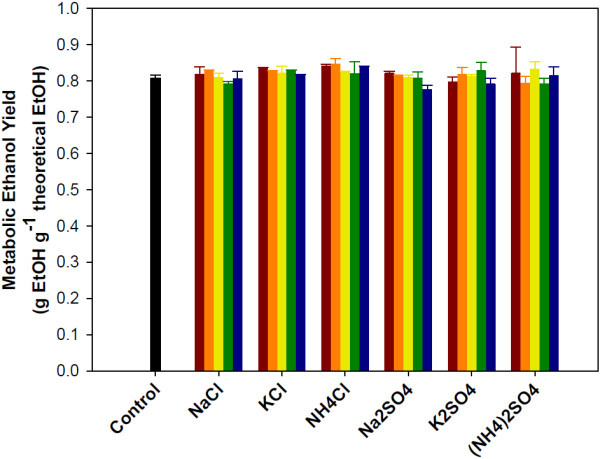
**Impact of salt on the metabolic ethanol yield during the co-fermentation of glucose and xylose by *S. cerevisiae. ***Salts concentration: no salt addition black bar, 0.1M red bar, 0.2M orange bar, 0.3M yellow bar, 0.4M green bar and 0.5M blue bar. Error bars represent standard errors. Experiments were carried out in duplicate.

**Figure 4:**
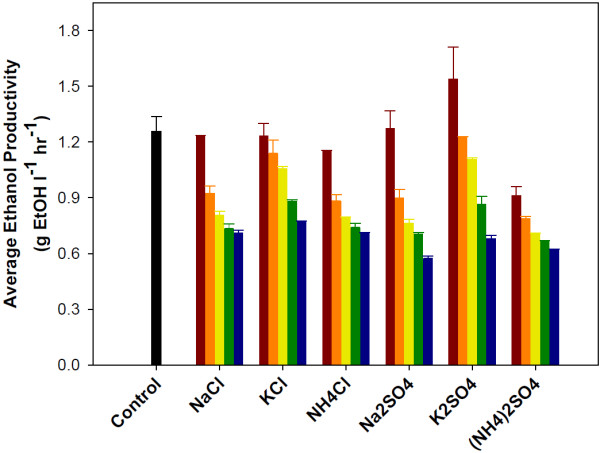
**Impact of salt on the average volumetric ethanol productivity during the co-fermentation of glucose and xylose by *S. cerevisiae*.** Salts concentration: no salt addition black bar, 0.1M red bar, 0.2M orange bar, 0.3M yellow bar, 0.4M green bar and 0.5M blue bar. Error bars represent standard errors. Experiments were carried out in duplicate.

To determine the effect of salts on ethanol production, metabolic yields and average volumetric production rates of ethanol were calculated (Figures 3 and 4). The ethanol metabolic yields were calculated by dividing the observed ethanol concentrations by the theoretical ethanol concentrations from the mass of sugar consumed by the yeast. The ethanol metabolic yield is used to determine fermentation efficiency; fermentation with all sugars converted to ethanol would have a metabolic yield of 1. In the presence of salts, the ethanol metabolic yields ranged from 0.77 to 0.84, while the control had a yield of 0.81. There was no consistent trend observed as salt concentration increased, rather the data shows minimal impact of salts on metabolic ethanol yield. ANOVA analysis of the data showed no significant differences (p < 0.05) in the yield caused by either salt type or salt concentration. Although the metabolic yield of ethanol is unchanged, the yields of glycerol and xylitol are affected by salt, as discussed below.

To determine if salts impacted the rate that yeast is able to convert sugars into ethanol, average ethanol volumetric productivities were calculated by dividing the maximum observed ethanol concentration by the fermentation time required to reach that concentration (Figure 4). This approach includes the potential effects of salts on both glucose and xylose fermentation. At a salts concentration of 0.1M (0.1M for cations paired with chloride and 0.2M for cations paired with sulfate), the only salt that resulted in a significant decrease in productivity was ammonium sulfate. However, a significant decrease in average ethanol volumetric productivity was observed with all salts at concentrations at or above 0.3M (0.6M cation concentration for sulfate salts). At a salts concentration of 0.5M (1M cation concentration for sulfate salts), the productivities were reduced by nearly 50% on average when compared to the control. This is a significant reduction in productivity and is not desirable from an economic viewpoint for industrial ethanol production. Ethanol productivity is directly linked to substrate consumption rates, since the metabolic yield of ethanol is not significantly affected by salts as discussed above. Therefore, overcoming salt inhibition requires reducing its effect on xylose consumption rate.

### Impact on intracellular metabolites

**Figure 5:**
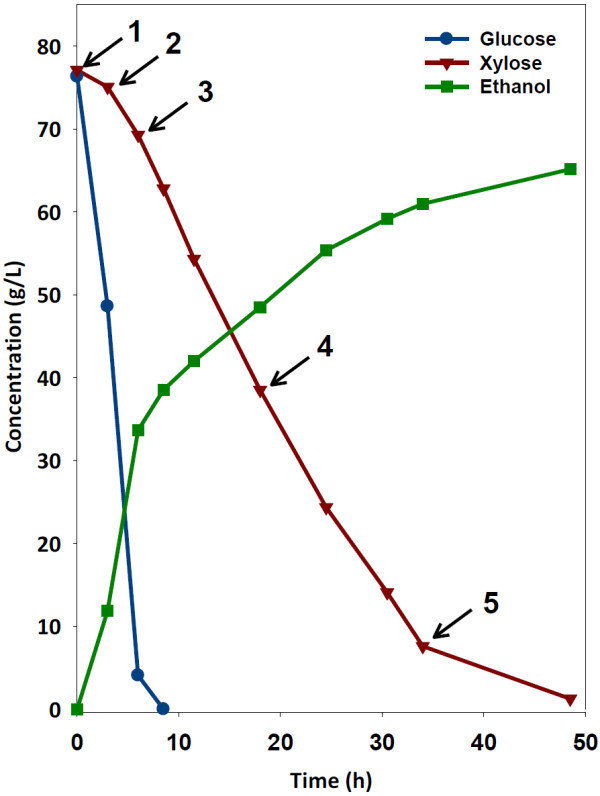
**Fermentation profile of control fermentation with sampling points.** Arrows designate time points for metabolomic analysis. The sampling points correspond to 1) beginning of primary glucose fermentation, 2) middle of primary glucose fermentation, 3) transition between primary glucose and primary xylose fermentation, 4) middle of primary xylose fermentation, and 5) end of fermentation. Glucose: ●, blue line; Xylose: ▼, red line; Ethanol: ■, green line.

To further explore the effect of salts on yeast metabolism, a metabolomic study was conducted. Intracellular metabolite concentrations for 19 metabolites, primarily those in the glycolytic and pentose phosphate pathways, were profiled throughout the control fermentation (no salt added) and fermentation containing 0.5M NaCl by taking samples at several points throughout the fermentation (Figure 5). Samples from two different fermentation conditions were analyzed: one set from a control fermentation with no salt and one set from a treatment fermentation with 0.5M NaCl. Because the rates of sugar consumption were different, samples in both treatments were taken when similar fermentation stages were reached (e.g. middle of glucose fermentation, and middle of xylose fermentation).

**Figure 6:**
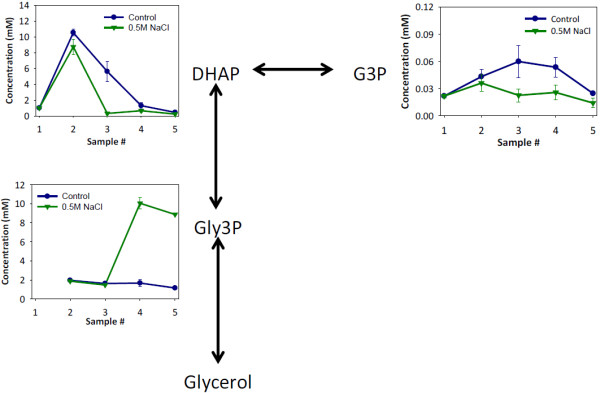
**Effect of 0.5M NaCl on glycerol pathway intracellular metabolite concentrations during the co-fermentation of glucose and xylose.** DHAP: dihydroxyacetone phosphate, G3P: glyceraldehyde 3-phosphate, Gly3P: glycerol 3-phosphate. Control fermentation: ●, blue line; Fermentation with 0.5M NaCl: ▼, green line. Error bars represent standard errors.

**Figure 7:**
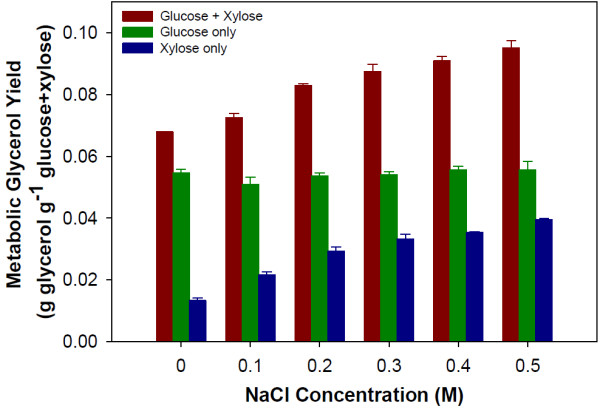
**Metabolic yields of glycerol observed during the co-fermentation of glucose and xylose by *S. cerevisiae *with the addition of NaCl.** Glucose + xylose red bar, glucose only green bar and xylose only blue bar. Error bars represent standard errors. Experiments were carried out in duplicate.

**Figure 8:**
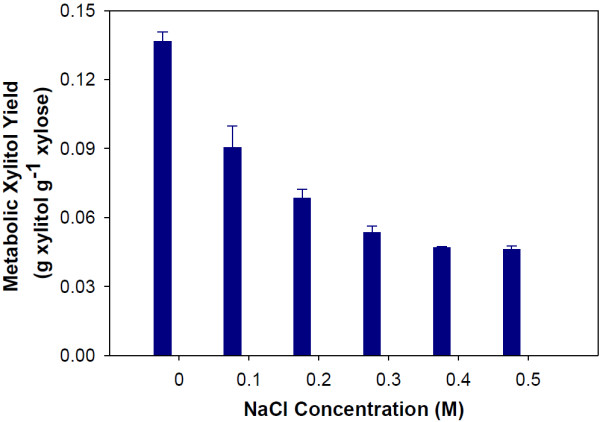
**Metabolic yields of xylitol from the fermentation of xylose by *S. cerevisiae *with the addition of NaCl.** Error bars represent standard errors. Experiments were carried out in duplicate.

Overall, the profiled metabolites were similar in concentration at the sample points between the two conditions. However, the profiles of some metabolites involved in the glycerol pathway did show significantly lower intracellular concentrations when NaCl was present (Figure 6). The major differences in glycerol pathway metabolite concentrations occur during time points 3–5, when the yeast are fermenting xylose as the sole carbon source. In the middle of xylose fermentation (sample 4) there is an almost 5-fold increase in glycerol 3-phosphate concentration when 0.5M NaCl is present compared to the control. At this time point, a decrease in glyceraldehyde 3-phosphate concentration is also observed. These results suggest that salt induces a change in flux toward glycerol production. It is widely known that one response to osmotic stress by yeast is increased production of glycerol [28]. Analysis of the yield of glycerol as a fraction of total xylose consumed support the intracellular metabolite concentration measurements. Glycerol yields increased as the concentration of NaCl increased (Figure 7). At 0.5M NaCl, the total glycerol yield from both glucose and xylose increased by 40% as compared to the control. When the carbon source of the glycerol is examined, nearly all of the increase can be attributed to xylose. As discussed above, metabolic yields of ethanol showed no change as a function of salt concentration. The increased production of glycerol from xylose is balanced by a concurrent decrease in the metabolic yield of xylitol (Figure 8). Therefore, carbon flux was shifted from xylitol to glycerol production when salt was present during xylose fermentation.

### Impact on cell growth

To determine the effect of salts on the growth of *S. cerevisiae* 424A(LNH-ST), the estimated growth rates from the logistic growth model were compared (data available in Additional file 1). The control fermentation (with no inhibitors present) had a growth rate constant of approximately 0.20 h^-1^. The inclusion of salt in the fermentation media reduced the growth rate for all salt types and concentrations tested. The minimum growth rate observed was 0.11 h^-1^ with 0.5M sodium sulfate (a decrease of 45% compared to the control). An approximate linear relationship was observed between growth rate and increasing salt concentration. Our results are consistent with findings described in literature that found salts have a negative impact on yeast growth when glucose is the sole fermentable sugar [16,17,29]. However, we should point out that under our experimental conditions there is only limited cell growth due to higher inoculum size. Thus effect of salt on cell growth using substantially lower inoculum could be different.

## Conclusions

The effect of inhibitors found in lignocellulose hydrolysis on yeast performance has been widely studied. However, few of these studies have investigated ions from salts, especially as they affect xylose fermentation. Studies of fermentation inhibition by salts examined the impact on yeast or bacterial strains fermenting glucose [16,17,30-32]. In this paper, we investigated the impact of salts on *S. cerevisiae* 424A(LNH-ST), a yeast strain capable of effectively fermenting both glucose and xylose. As expected, cell growth rate, glucose utilization, and ethanol productivity decreased with high salt concentrations. In addition, the data showed that xylose utilization is affected by salts present at any concentration with severe reduction of xylose consumption rate at higher salt concentrations. Quantification of intracellular metabolites and a carbon balance around xylose metabolism showed increased metabolic flux to glycerol, a known osmoprotectant, at expense of xylitol production during xylose fermentation. This suggests that yeast strains are more susceptible to osmotic stress in the presence of salts when xylose is the sole carbon source. This may be due in part to the reduced ATP generation rate and yield during fermentation of xylose when compared to glucose. A previous study showed that *Saccharomyces* yeast was able to overcome this energy limitation and reduce the inhibitory effect of acetic acid on xylose fermentation by feeding glucose at low concentrations to restore ATP levels [21]. A similar approach may mitigate the impact of salt on xylose fermentation.

Of the ion pairs tested, the greatest variability in inhibition was between different cations paired with the same anion, in contrast to different anions paired with the same cation. Potassium salts had the least inhibitory effect of the salts tested. These results imply that chemicals that generate potassium salts should be used in place of chemicals that produce sodium or ammonium salts in lignocellulose conversion processes in order to minimize salt inhibition during fermentation.

## Materials and methods

### Yeast strain

*Saccharomyces cerevisiae* 424A(LNH-ST), a recombinant yeast strain capable of the co-fermentation of glucose and xylose, was utilized in the experiments [20,22,33].

### Fermentation experiments

To prepare the inoculum, 2 ml of seed culture was used to inoculate 100 ml YEPD media [1% yeast extract, 2% peptone, 2% glucose] (Mallinckrodt Chemicals, Phillipsburg, NJ) in 300 ml baffled sidearm Erlenmeyer flasks (Bellco, Vineland, NJ). The culture was incubated aerobically overnight in a shaker set at 28°C and 200 rpm.

Micro-aerobic batch fermentations were performed in 300 ml baffled sidearm Erlenmeyer flasks [34]. To prepare the flasks for fermentation, appropriate amounts of glucose, xylose, and salts were added to 100 ml YEP media to result in the desired concentration of each. Glucose and xylose concentrations were set at 70 g/L each. For the xylose only fermentation, initial xylose concentration was set to 140 g/L. The salts examined were NaCl, KCl, NH_4_Cl, Na_2_SO_4_, K_2_SO_4_, and (NH_4_)_2_SO_4_ at concentrations ranging from 0.1 to 0.5M.

Once the cell density of the growth culture reached approximately 500 KU (6 g dry cells/L), the culture was harvested by centrifugation for 5 minutes at 3100 × g. The cell pellet was resuspended in YEP and this mixture was used to inoculate the fermentation flasks to an initial cell density of 400 KU (4.75 g dry cells/L). The flasks were sealed with plastic wrap to result in micro-aerobic fermentation conditions and transferred to an orbital shaker set at 28°C and 200 rpm. The fermentation then proceeded for 72 hours. All fermentations were carried out in duplicate.

### Analysis of fermentation substrates and products

For the analysis of substrate consumption and product formation, 1 ml of the fermentation broth was collected throughout the fermentation and centrifuged for 5 minutes at 9000 × g. The resulting supernatant was collected and stored at −20°C until further analysis.

The fermentation metabolites were analyzed by HPLC using the method outlined by Lu et al. [23] using a Waters Alliance 2695 HPLC system with an Aminex® HPX-87H 300 × 7.8 mm column (Bio-Rad Laboratories, Hercules CA). The HPLC column operating conditions were 60°C and a flow rate of 0.6 ml/minute for the mobile phase, 5 mM sulfuric acid in distilled, deionized water.

### Calculation of substrate consumption rates

An unstructured model was used to analyze the fermentation data and calculate the specific substrate consumption rates for both glucose and xylose with units of grams of sugar consumed per gram of cell (dry weight) per hour. A detailed description of the model is described by Casey et al. [35].

### Analysis of intracellular metabolites

For the fermentation with 0.5M NaCl, intracellular metabolite analysis was also conducted. Samples for metabolomic analysis were collected at different significant metabolic stages during the fermentation time course. Two biological replicates were collected at each time point, and the process was repeated to get technical duplicates, resulting in a total of 4 samples per time point. Sampling and metabolite extraction was performed as described by Gonzales et al. [36] and Lange et al. [37]. Simultaneous quantification of glycolytic and pentose phosphate pathway metabolites was done according Yang et al. [38,39] using reversed-phase liquid chromatography-mass spectrometry and in vitro ^13^C labeling. We determined the concentration of19 metabolites (glucose-6-phosphate, 6-phosphogluconate, ribulose-5-phosphate, erythrose-4-phosphate, ribose-5-phosphate, xylulose-5-phosphate, xylulose, fructose-6-phosphate, fructose 1,6-bisphosphate, glyceraldehyde-3-phosphate, dihydroxyacetone-phosphate, glycerol- 3-phosphate, glycerate 1,3-bisphosphate, 3- phosphoglycerate, phospho(enol)pyruvate, trehalose, ATP, ADP, AMP).

## Abbreviations

ANOVA: Analysis of variance; HPLC: High performance liquid chromatography; KU: Klett unit; DHAP: Dihydroxyacetone phosphate; G3P: Glyceraldehyde 3-phosphate; Gly3P: Glycerol 3-phosphate; EtOH: Ethanol.

## Competing interests

The authors declare that they have no competing interests.

## Authors’ contributions

EC compiled and analyzed the fermentation data, completed half of the metabolomics study, and drafted the manuscript. NSM reviewed the results and edited the manuscript. JA designed the metabolomics analysis method and oversaw the analysis of the samples. ZS completed half of the metabolomics study. NH reviewed the fermentation and made helpful suggestions to the manuscript. MS designed, led and coordinated the overall study. All authors have read and approved the final manuscript.

## Supplementary Material

Additional file 1 Modeling the Effect of Acetic Acid on Batch Co-Fermentations of Glucose/Xylose to Ethanol by Saccharomyces cerevisiae 424A(LNH-ST). Click here for file
