# Supplementary material for: Effect of salts on the Co-fermentation of glucose and xylose by a genetically engineered strain of Saccharomyces cerevisiae
Source: Biotechnol Biofuels. 2013 May 29;6:83. doi: 10.1186/1754-6834-6-83 (PMC3671970; doi:10.1186/1754-6834-6-83)
Supplement: Additional file 1 — Modeling the Effect of Acetic Acid on Batch Co-Fermentations of Glucose/Xylose to Ethanol by Saccharomyces cerevisiae 424A(LNH-ST). [file 1754-6834-6-83-S1.pdf]

Modeling the Effect of Acetic Acid on Batch Co-Fermentations of Glucose/Xylose to Ethanol by Saccharomyces cerevisiae 424A(LNH-ST)

Journal:	*Biotechnology and Bioengineering*
Manuscript ID:	Draft
Wiley - Manuscript type:	Article
Date Submitted by the Author:	n/a
Complete List of Authors:	Casey, Elizabeth; Purdue University, ABE/LORRE<br>Clingenpeel, Shane; Purdue University, ABE/LORRE<br>sedlak, mira; Purdue University, ABE/LORRE<br>Ho, Nancy; Purdue University, LORRE<br>Mosier, Nathan; Purdue University, LORRE
Key Words:	Kinetic model, cellulose, ethanol, acetic acid, xylose, yeast

SCHOLARONE™
Manuscripts

For Peer Review

Modeling the Effect of Acetic Acid on Batch Co-Fermentations of Glucose/Xylose to Ethanol by *Saccharomyces cerevisiae* 424A(LNH-ST)

by

Elizabeth Casey[1,2], Shane Clingenpeel[1,2], Miroslav Sedlak[1,2], Nancy W. Y. Ho[1,3], Nathan Mosier[1,2]

1. Laboratory of Renewable Resources Engineering, Purdue University
2. Department of Agricultural and Biological Engineering, Purdue University
3. Department of Chemical Engineering, Purdue University
West Lafayette, IN 47907

Running Title: Modeling Glucose/Xylose Cofermentation

Abstract

A kinetic model for the batch co-fermentation of glucose and xylose to ethanol by *S. cerevisiae* 424A(LNH-ST) is reported. Experimental data was used to develop an unstructured model that includes cell growth, glucose consumption, xylose consumption, ethanol production, glycerol production, and xylitol production. Good agreement between the model predicted values and experimental data was observed for basic fermentations without inhibitory compounds present. Additional model parameters were required to account for the inhibition caused by acetic acid. Interpretation of the estimated model parameters provides insight into the fermentative performance of the microorganism under the tested conditions. For the strain tested in this study, *S. cerevisiae* 424A(LNH-ST), the xylose consumption rate was one third that of glucose under normal conditions and almost eight times slower when acetic acid was present. This highlights potential target area for strain improvement. The methodology developed here can be used to interpret fermentation results and estimate performance parameters critical for process optimization and scale-up.

Keywords

Kinetic model, acetic acid, ethanol, xylose fermentation, yeast

For Peer Review

INTRODUCTION

Efficient conversion of cellulosic biomass to ethanol via biochemical processing requires microorganisms that are tolerant to inhibitors and products, consume a wide-range of substrates (both hexose and pentose sugars), and have high productivity to result in high yield (Aristidou, 2007). Significant progress has been made on the development of organisms capable of mixed sugar fermentation (See Weber et al., 2010 for review). Work is ongoing to further improve the ability of organisms to efficiently ferment multiple substrates to ethanol in the presence of inhibitors (See Parawira and Tekere, 2011 for review).

Evaluation of strain improvements should be approached two ways: 1) comparison of fermentation performance characteristics (cell growth, substrate utilization, and product formation) between the original and improved strain to determine the degree and type of improvements and 2) how those improvements impact commercialization. Both approaches require estimation of important fermentation process parameters. A mathematical model of the fermentation process is an efficient way to determine these parameters and can be a valuable tool for researchers in the area of organism development. A fermentation model able to predict performance over a wide range of conditions must include the effect of process conditions such as temperature and pH as well as concentrations of cells, substrate(s), and product(s). One application of a model is to interpret experimental results. For example, the data resulting from a typical fermentation experiment is the concentration of cells, substrates, and products at specific time points during the fermentation. A model allows a researcher to easily estimate fermentation performance parameters such as specific growth rate or specific substrate consumption rate from that data. They could then quantitatively compare how changes to the fermentation process (e.g. different strains of a microorganism or presence of inhibitors) affect fermentation performance.

For Peer Review

These performance parameters are also needed to guide the scale-up and design of commercial processes. Another important application for a model is to predict how the process will be impacted by changes in conditions. For example, how will the fermentation respond to a reduced initial cell density, a different pH, or a different ratio of substrates? This could significantly reduce the amount of time and money required to design and optimize a process by limiting the amount of experimental work to focus on processing parameters with the greatest impact on performance, e.g. product yield and process productivity.

The steps required for the development of a model for a process such as fermentation have been clearly outlined (Mavituna and Sinclair, 2008). The basic approach is to define the system to be modeled, identify variables of interest, develop equations relating the variables, estimate model parameters with experimental data, validate model with additional data, refine model as needed with additional experiments, and use model for desired application(s). The fermentation of glucose to ethanol by *S. cerevisiae* has been widely modeled using unstructured, Monod-like kinetics (Maiorella et al. 1984a; Maiorella et al. 1984b; Monod 1949). However, the addition of a xylose fermentation pathway to yeast requires that the well-established fermentation kinetic models be expanded. There have been previous reports of models for glucose/xylose co-fermentation in *Saccharomyces cerevisiae* (Krishnan et al., 1999), *Escherichia coli (*Olsson and Hahn-Hagerdal, 1995), and *Zymomonas mobilis* (Leksawasdi et al., 2001). Each of these models incorporated xylose fermentation by first developing the rate equations for glucose and xylose fermentation separately using Monod-type kinetics. The models were then extended to account for mixed glucose and xylose fermentation. Although the resulting models successfully predicted mixed sugar fermentation performance, each has specific characteristics that make them inappropriate or incomplete for the present study and its' desired application.

For Peer Review

The *S. cerevisiae* and *Z. mobilis* models were developed using Monod kinetics for cell growth; however, the growth conditions of the present study are not well modeled with this approach. The *E. coli* model did not account for cell growth, instead assuming a constant cell concentration over time. The *S. cerevisiae* and *E. coli* models were designed so that the primary fermentation performance characteristics estimated were cell growth and ethanol production. Substrate consumption was then related to ethanol production using a yield coefficient. However, previously reported experimental data suggests that the yield coefficients vary between glucose and xylose and are affected by the presence of inhibitors (Casey et al., 2010). Therefore substrate equations that are independent of ethanol yield are required. None of the models evaluated byproduct formation (e.g. glycerol, xylitol), an important but often overlooked performance characteristic. Previous research has shown byproduct formation to vary when inhibitors such as salts are present (Casey et al., 2012).

In the present study, the kinetics of the simultaneous co-fermentation of glucose and xylose to ethanol by *S. cerevisiae* 424A(LNH-ST), a recombinant industrial yeast strain capable of xylose fermentation, were investigated. The experimental results were used to develop an unstructured fermentation model of cell growth, glucose and xylose consumption, ethanol production, and glycerol and xylitol production.

Materials and Methods

Yeast Strain

All fermentations utilized *Saccharomyces cerevisiae* 424A(LNH-ST), a recombinant yeast strain capable of the co-fermentation of glucose and xylose (Ho et al., 2000; Ho et al., 1998).

Batch Fermentation Experiments

For Peer Review

A number of micro-aerobic batch fermentations with varying conditions were conducted. All fermentations were completed in 1 liter New Brunswick BioFlo 110 benchtop fermentors (Edison, NJ) equipped with pH control. The inoculum for the fermentor was prepared by pre-growing yeast aerobically in a shaker set at 28°C and 200 rpm in 2 L flasks containing 500 mL YEPD media [1% yeast extract, 2% peptone, 2% glucose] (Mallinckrodt Chemicals, Phillipsburg, NJ).

Once the cell density of the growth culture reached approximately 6 g dry cells L^{-1}, the culture was centrifuged for 5 minutes at 14000 rpm. The cell pellet was re-suspended in 10 mL YEP [1% yeast extract, 2% peptone] and used to inoculate a fermentor containing 800 mL YEP media plus appropriate amounts of glucose and xylose at pH 5.5. The initial cell density ranged from 0.6 to 4.75 g dry cell L^{-1}. The temperature and agitation of the fermenter was maintained at 28°C and 200 rpm, respectively. The media pH was continuously controlled within ±0.01 of 5.5 using 1M phosphoric acid and 1M ammonium hydroxide.

Sampling and Analysis of Fermentation Substrates and Products

To monitor the cell growth during fermentation, cell density was measured at various points throughout the fermentation time course using a Klett colorimeter (Manostat Co., New York, NY). Calibration of measurements from the Klett colorimeter to cell dry mass (g L^{-1}) for this strain was performed as previously described (Bera et al., 2010). Also at each time point, 1 mL of fermentation broth was collected for the analysis of substrate and product concentration. The sample was centrifuged for 5 minutes at 14000 rpm and the resulting supernatant was collected and stored at -20°C until further analysis.

For Peer Review

Samples were analyzed for glucose, xylose, glycerol, xylitol, acetic acid, and ethanol by HPLC using the method outlined by Lu et al. (2009) with a Waters Alliance 2695 HPLC system with an Aminex® HPX-87H 300 x 7.8 mm column (Bio-Rad Laboratories, Hercules CA). The HPLC column operating conditions were 60°C and a flow rate of 0.6 ml min^{-1} for the mobile phase, 5 mM sulfuric acid in water.

MODEL DEVELOPMENT

An unstructured fermentation model was developed to depict the batch fermentation of glucose and xylose to ethanol by *S. cerevisiae* 424A(LNH-ST). The model includes expressions for cell growth, substrate consumption (glucose and xylose), product formation (ethanol), and byproduct formation (glycerol and xylitol).

Cell Growth

To model the cell density throughout the entire fermentation, a classical logistic growth expression was used (1) (Pearl, 1927). A Levenspiel inhibition term was included to account for cell growth inhibition by ethanol (Levenspiel, 1980).

$$\frac{d[C]}{dt} = \mu * C * \left(1 - \frac{C}{C_{max}}\right) * \left(1 - \frac{P}{P_{max,grow}}\right)^n \quad \textbf{(1)}$$

Where C is cell density (g dry cell weight l^{-1}), C_{max} is the maximum cell density (g dry cell weight l^{-1}), μ is the growth rate (h^{-1}), P is ethanol concentration (g ethanol l^{-1}), $P_{max,grow}$ is the maximum tolerable ethanol concentration for cell growth, and n is an exponential inhibition factor. For cell growth, $P_{max,grow}$ has been estimated at 87 g L^{-1} and n estimated at 1 (Ghose and Tyagi, 2004).

Substrate Consumption

For Peer Review

A reverse approach of that from the published glucose/xylose fermentation models (Krishnan et al., 1999; Olsson and Hahn-Hagerdal, 1995; Leksawasdi et al., 2001) was used to model substrate consumption. Substrate consumption was modeled as a function of cell concentration with ethanol production accounted for using yield coefficients. This is possible because ethanol fermentation is considered a Type I process where substrate consumption is directly related to the production of the main product (Mosier and Ladisch, 2009). A reconfiguration of the product formation model by Maiorella et al. (1984a) was used to create a substrate consumption model that also includes the Levenspiel product inhibition term (2).

$$\frac{dS}{dt} = -\frac{v_{max} * S}{K_m + S} * \left(1 - \frac{P}{P_{max}}\right)^n * C \qquad \textbf{(2)}$$

Where S is substrate concentration, v_{max} is the maximum specific substrate consumption rate, and K_m is the substrate concentration when the specific consumption rate is half its maximum.

This generic model can be applied to both glucose and xylose consumption. For glucose consumption, Equation (2) can be simplified to Equation (3) because the K_m value for glucose of 0.315 g L^{-1} (Maiorella et al., 1984a) is negligible compared to the glucose concentrations observed over the course of a batch fermentation.

$$\frac{dG}{dt} = -v_{max,g} * C * \left(1 - \frac{P}{P_{max,g}}\right)^n \qquad \textbf{(3)}$$

Where G is glucose concentration (g l^{-1}), $v_{max,g}$ is the maximum glucose consumption rate (g glucose g^{-1} cells h^{-1}), and $P_{max,g}$ is the maximum tolerable ethanol concentration for glucose consumption. For glucose consumption, $P_{max,g}$ is estimated at 140 g L^{-1} for the studied strain (unpublished data). This is significantly higher than $P_{max,grow}$. As has been reported in the literature, ethanol inhibits the growth of *S. cerevisiae* more strongly than fermentation of glucose

For Peer Review

(Brown et al., 1981). *S. cerevisiae* is able to continue fermenting sugars to ethanol after growth is inhibited.

For xylose consumption an additional term must be added to Equation (2) to model the delay in xylose consumption when glucose is present (Figure 1). It is evident in Figure 1 that xylose consumption is minimal until the glucose concentration approaches zero. This delay is thought to be caused by a higher affinity by the transporter proteins for glucose compared to xylose. For example, the binding constant of glucose to a major glucose transporter, HXT7, is approximately 1 mM (Reifenberger et al., 1997), while the binding constant for xylose is approximately 130 mM, a difference of two orders of magnitude (Saloheimo et al., 2007). Therefore, glucose acts a competitive inhibitor to the uptake of xylose (Lee et al., 2002; Bertilsson et al., 2008). To model this inhibition, a competitive inhibition term is added. The resulting xylose consumption model is provided in Equation (4).

$$\frac{dX}{dt} = -\frac{v_{max,x} * X * C}{K_{m,x} * (1 + \frac{G}{G_i}) + X} * \left(1 - \frac{P}{P_{max,x}}\right)^n \quad \textbf{(4)}$$

Where X is xylose concentration (g L^{-1}), $v_{max,x}$ is the maximum xylose consumption rate (g xylose g^{-1} cells h^{-1}), and $K_{m,x}$ is the xylose concentration (g L^{-1}) when the consumption rate is half its maximum. G_i is the glucose inhibition constant (g L^{-1}) and was estimated at 10 g L^{-1} by determining the glucose concentration at which significant xylose consumption was first observed (Figure 1). $K_{m,x}$ has been previously estimated as 16.7 g l^{-1} (Athmanathan et al. 2011). $P_{max,x}$ has been previously determined to be 121 g $l^{-1}$with an n of 1 for *S. cerevisiae* 424A(LNH-ST) (Athmanathan et al., 2011). $P_{max,x}$ is lower than $P_{max,g}$, indicating that ethanol is more inhibitory to xylose fermentation than glucose fermentation. Athmanathan et al. (2011) also reported that $P_{max,x}$ is affected by the presence of glucose in the fermentation medium. This strain

For Peer Review

of *S. cerevisiae* is better able to tolerate ethanol while fermenting xylose if glucose was initially present than if xylose is fermented alone, even though the majority of xylose is consumed after the glucose is depleted from the medium. It was hypothesized that physiological changes made during the glucose fermentation phase enabled the cells to better tolerate ethanol when xylose was the sole carbon source.

Product Formation

The Type I substrate/product relationship allows the use of a yield coefficient to model the production of ethanol. Two separate yield coefficients are estimated: one for ethanol from glucose and one for ethanol from xylose. Ethanol yields have been observed to vary between substrates in glucose/xylose fermenting microorganisms (Krishnan et al., 1999). The resulting expression is provided in Equation (5).

$$\frac{dP}{dt} = -Y_{P/G} * \frac{dG}{dt} - Y_{P/X} * \frac{dX}{dt} \quad \textbf{(5)}$$

Where P is ethanol concentration (g L^{-1}), $Y_{P/G}$ is the yield coefficient of ethanol formed per glucose consumed (g ethanol g^{-1} glucose), and $Y_{P/X}$ is the yield coefficient of ethanol formed per xylose consumed (g ethanol g^{-1} xylose).

Byproduct Formation

As with ethanol production, the production of the byproducts glycerol and xylitol is directly related to substrate consumption; therefore yield coefficients can also be used to model byproduct formation. Glucose is converted to ethanol by *S. cerevisiae* 424A(LNH-ST) through the Embden-Meyerhof-Parnas pathway. Xylose is converted to ethanol by *S. cerevisiae* 424A(LNH-ST) in four main steps: 1) conversion of xylose to xylulose, 2) phosphorylation of xylulose to xylulose 5-phosphate, 3) conversion of xylulose 5-phosphate to glycolytic intermediates through the pentose phosphate pathway, and 4) Embden-Meyerhof-Parnas

For Peer Review

glycolysis (Ho et al., 2000). Glycerol is produced from the glycolytic intermediate dihydroxyacetone phosphate and xylitol is produced as an intermediate in the conversion of xylose to xylulose. Therefore glycerol can result from the consumption of both glucose and xylose, so two separate yield coefficients are used (6). Xylitol results only from the consumption of xylose, so a single yield coefficient is used (7).

$$\frac{dGly}{dt} = -Y_{Gly}/_{G} * \frac{dG}{dt} - Y_{Gly}/_{X} * \frac{dX}{dt} \quad \textbf{(6)}$$

$$\frac{dXyl}{dt} = -Y_{Xyl}/_{X} * \frac{dX}{dt} \quad \textbf{(7)}$$

Where *Gly* is glycerol concentration (g L^{-1}), $Y_{Gly/G}$ is the yield coefficient of production of glycerol formed per glucose consumed (g glycerol g^{-1} glucose), $Y_{Gly/X}$ is the yield coefficient of production of glycerol formed per xylose consumed (g glycerol g^{-1} xylose), *Xyl* is xylitol concentration (g L^{-1}), and $Y_{Xyl,X}$ is the yield coefficient of production of xylitol formed per xylose consumed (g xylitol g^{-1} xylose).

Parameter Estimation

The system of ordinary differential equations (ODEs) described above were numerically integrated using the Euler method with a 0.5 hour time step in Microsoft Excel ®. The Solver tool in 2007 Microsoft Excel ® was utilized to estimate the parameters by minimizing residual sum of squares (RSS) between the model predicted values and experimental values. This tool utilizes the Generalized Reduced Gradient Algorithm for optimization.

RESULTS AND DISCUSSION

To estimate the parameters in the model, the data from the fermentations with initial concentrations of 60 g L^{-1} glucose, 60 g L^{-1} xylose, and 4.75 g dry cells L^{-1} was used. To minimize potential errors in the experimental data, the results from three replicate experiments

For Peer Review

were averaged. Figure 2 presents the averaged experimental values along with the associated model predicted curves. The optimized fermentation performance parameters associated with this figure are provided in Table 1.

Visually, the model predicted values appear to be in good agreement with the experimental data. To better assess the fit of the model to the experimental data, the RSS and coefficient of determination (R^2) were examined (Table 2). R^2 was calculated by subtracting the ratio of the RSS to total sum of squares from 1. The total RSS for the model was 47.89. Of the previously reported models for glucose and xylose fermentation, only one paper reported their RSS after model optimization. Their values ranged from 15.9 to 156 depending on the fermentation conditions (Leksawasdi et al., 2001). Our RSS falls within the lower end of this range. All correlation coefficients were greater than 0.95, showing an excellent fit of the model to the experimental data.

To validate the model, a series of fermentations were conducted. Different glucose and xylose concentrations, as well as different initial cell densities, were tested to evaluate how the model performs under a variety of initial fermentation conditions. Statistical analysis of the degree of fit of the model to the observed experimental values for the different fermentations is provided in Table 3. Overall, there is excellent agreement between the model with reduced initial sugar concentrations and/or reduced initial cell mass. For the reduced initial sugar concentration, the fit of the cell mass model had a low R^2. To explore this, the growth rate parameter was re-optimized for the reduced sugar fermentation. The new growth rate was 0.09 h^{-1}, compared to the original growth rate of 0.22 h^{-1}. This suggests that growth rate may be dependent on sugar concentration, which would require refinement of the cell growth ODE. Further sensitivity analysis is needed to confirm this, requiring additional fermentations over a wide range of initial

For Peer Review

substrate concentrations. For the reduced initial cell density fermentation, all model components showed excellent fit with the original estimated model parameters. These results validate that the model accurately predicts fermentation performance of *S. cerevisiae* 424A(LNH-ST) over a range of initial conditions.

Having developed the preliminary model, some conclusions can be drawn regarding the fermentation performance of this strain of glucose/xylose co-fermenting *S. cerevisiae*. There was a significant difference in substrate consumption rate between the two sugars. The consumption of glucose was about three times faster than xylose. The ethanol yield was 70% of theoretical from glucose and 80% of theoretical from xylose. Part of this difference in ethanol yield could be related to the fact that cell growth occurs primarily during glucose fermentation. A mass balance was conducted around each substrate accounting for all measured products (ethanol + CO_2, glycerol, xylitol, and cell mass where 1 g of cells is results from 1.25 g of consumed substrate, based on carbon content (Wang et al., 1979)). These products account for 84% of the consumed glucose and 94% of the consumed xylose.

The model can also be used to compare fermentation performance when experimental conditions are altered. An example would be the addition of an inhibitor to the fermentation media. Acetic acid has been shown to be inhibitory to fermentative microorganisms. The optimized model was applied to data from a fermentation with identical conditions as the calibration data, with the exception of the addition of acetic acid (10 g L^{-1}) to the initial fermentation media. The fit results are provided in Table 4. The original model provides a good fit for the cell mass, glucose, and ethanol components, but xylose and the byproducts are poorly modeled. This suggests that acetic acid has an impact on the consumption of xylose and production of glycerol and xylitol. It has been shown previously that acetic acid is extremely

For Peer Review

inhibitory to xylose consumption (Casey, Sedlak, et al. 2010). Therefore, the model the addition of an inhibition term in the xylose consumption ODE to account for the effect of acetic acid. Prior research reports xylose consumption rate as a function of acetic acid concentration and media pH (Casey, 2008). Equation 4 was modified to include the relationship between pH and acetic acid concentration on xylose consumption.

$$\frac{dX}{dt} = -\frac{v_{max,x} * X * C}{K_{m,x} * (1 + \frac{G}{G_i}) + X} * \left(1 - \frac{P}{121}\right)^1 * e^{-0.5765*\frac{AA}{10^{pH-pKa}+1}} \quad \textbf{(8)}$$

Where AA is the total acetic acid and acetate concentration (g L^{-1}), pH is the media pH, pK_a is the association constant for acetic acid (4.75), and 0.5765 is the inhibition constant for acetic acid for the given strain (Casey, 2008). The resulting fit of the model to experimental data is included in Table 4. The model fit toward xylose consumption is greatly improved when acetic acid inhibition is modeled, but the byproducts are still poorly modeled. The ethanol model also now shows a poor fit. To improve the fit, the parameters for the ethanol and byproduct models were re-optimized. The new fit results are shown in Table 4 and the new parameters are shown in Table 5. The updated model shows a good fit.

The inhibition term and re-optimized parameters provide insight into how acetic acid affects fermentation performance of *S. cerevisiae* 424A(LNH-ST). To understand the impact of acetic acid on xylose consumption, the inhibition term was calculated for the given experimental conditions (10 g l^{-1} AA and pH 5.5). The resulting number was 0.42 meaning acetic acid reduces the xylose consumption rate 42% compared to the same conditions without acetic acid present. This inhibition term is useful for process optimization because it allows you to estimate how changing the pH or including processing steps such as acetic acid removal can impact fermentation performance. However, an inhibition term may not be mathematically defined for

For Peer Review

all inhibitors. An alternative application of the model to analyze inhibition is to re-optimize the fermentation parameter itself and compare to the control. This approach was usd to understand the effect of salts on fermentation performance of *S. cerevisiae* 424A(LNH-ST) (Casey et al., 2012).The re-optimized ethanol parameters show an increase in yield when acetic acid is present. This finding is consistent with previous reports and explained by the diversion of carbon from cell mass to ethanol production for increased ATP generation (Casey et al., 2010; Taherzadeh et al., 1997). The parameters also indicate that the yield of glycerol from glucose decreases by more than 40% while the glycerol yield from xylose doubles. However, total glycerol produced from both substrates decreases by 16**%** in the presence of acetic acid in comparison to the control. The estimated yield of xylitol also decreased from 0.118 g g^{-1} to 0.038 g g^{-1} in the presence of acetic acid, a reduction of 68%. Mass balances around the consumed sugars had closures of 96% for glucose and 95% for xylose. The primary reason for the better closure around glucose in the acetic acid case compared to the control was that the yield of ethanol was higher (0.44 g g^{-1} versus 0.35 g g^{-1}).

CONCLUSIONS

In this study, an unstructured model of the co-fermentation of glucose and xylose to ethanol by *S. cerevisiae* 424A(LNH-ST) was developed. Using experimental data, model parameters were estimated to predict cell growth, substrate consumption (glucose and xylose), product formation (ethanol), and byproduct formation (glycerol and xylitol). The model fitted curves were in good agreement with the experimental results for fermentations without inhibitors present. Further model development was required when the inhibitor acetic acid was included to account for negative impact acetic acid has on the fermentation performance of *S. cerevisiae* 424A(LNH-ST). Potential applications for this model include determination of important process

For Peer Review

parameters such as substrate consumption rates and product/byproduct yield coefficients and comparison of these parameters for different experimental conditions or microorganisms.

ACKNOWLEDGMENTS

This work is supported by the US Department of Energy Biomass Program, Contract GO17059-16649 and Purdue Agricultural Research Programs. This material is partially based upon work supported under a National Science Foundation Graduate Research Fellowship. Any opinions, findings, conclusions or recommendations expressed in this publication are those of the authors and do not necessarily reflect the views of the National Science Foundation. The authors thank John Schumm for the preliminary modeling work he completed on this project and Xingya (Linda) Liu for conducting the HPLC analyses.

For Peer Review

References

Aristidou AA. 2007. Application of Metabolic Engineering to the Conversion of Renewable Resources to Fuels and Fine Chemicals: Current Advance and Future Prospects. In: El-Mansi EMT, editor. Fermentation Microbiology and Biotechnology. Boca Raton: CRC Press.

Athmanathan A, Sedlak M, Ho NWY, Mosier NS. 2011. Effect of Product Inhibition on Xylose Fermentation to Ethanol by Saccharomyces cerevisae 424A (LNH-ST). Biological Engineering Transactions 3(2):111-124.

Bera AK, Sedlak M, Khan A, Ho NW. 2010. Establishment of L-Arabinose Fermentation in Glucose/Xylose Co-Fermenting Recombinant *Saccharomyces cerevisiae* 424A(LNH-ST) by Genetic Engineering. Applied Microbiology and Biotechnology 87(5): 1803-1811.

Bertilsson M, Andersson J, Lidén G. 2008. Modeling Simultaneous Glucose and Xylose Uptake in Saccharomyces cerevisiae from Kinetics and Gene Expression of Sugar Transporters. Bioprocess and Biosystems Engineering 31(4): 369-377.

Brown SW, Oliver SG, Harrison DEF, Righelato RC. 1981. Ethanol Inhibition of Yeast Growth and Fermentation: Differences in the Magnitude and Complexity of the Effect. European Journal of Applied Microbiology and Biotechnology 11: 151-155.

Casey E. 2008. Effect of Acetic Acid Under Controlled pH Conditions on the Co-Fermentation of Glucose and Xylose by *Saccharomyces Cerevisiae* 424A(LNH-ST). (Master's thesis). Retrieved from ProQuest Dissertations and Theses. (Publication No. 1469631).

Casey E, Sedlak M, Ho NW, Mosier NS. 2010. Effect of Acetic Acid and pH on the Cofermentation of Glucose and Xylose to Ethanol by a Genetically Engineered Strain of *Saccharomyces cerevisiae*. FEMS Yeast Research 10: 385-393.

Casey E, Mosier NS, Ho NW, Sedlak, M. 2012. Effect of Salts on the Co-Fermentation of Glucose and Xylose by a Genetically Engineered Strain of *Saccharomyces cerevisiae* 424A(LNH-ST). Manuscript submitted for publication.

Ghose TK, Tyagi RD. 2004. Rapid Ethanol Fermentation of Cellulose Hydrolysate. II. Product and Substrate Inhibition and Optimization of Fermentor Design. Biotechnology and Bioengineering 21(8): 1401-1420.

Ho NWY, Chen Z, Brainard AP, Sedlak M. 2000. Genetically engineered *Saccharomyces* yeasts for conversion of cellulosic biomass to environmentally friendly transportation fuel ethanol. In: Anastas PT, Heine LG, Williamson TC, editors. ACS Symposium Series 767. Washington, DC: American Chemical Society. p 143-159.

Ho NWY, Chen ZD, Brainard AP. 1998. Genetically engineered Sacccharomyces yeast capable of effective cofermentation of glucose and xylose. Applied and Environmental Microbiology 64(5):1852-1859.

For Peer Review

Krishnan MS, Ho NWY, Tsao GT. 1999. Fermentation Kinetics of Ethanol Production from Glucose and Xylose by Recombinant *Saccharomyces* 1400(pLNH33). Applied Biochemistry and Biotechnology 78(1-3): 373-388.

Lee WJ, Kim MD, Ryu YW, Bisson LF, Seo JH. 2002. Kinetic Studies on Glucose and Xylose Transport in *Saccharomyces cerevisiae*. Applied Microbiology and Biotechnology 60(1): 186-191.

Leksawasdi N, Joachimsthal EL, Rogers PL. 2001. Mathematical Modeling of Ethanol Production from Glucose/Xylose Mixtures by Recombinant *Zymomonas mobilis*." Biotechnology Letters 23: 1087-1093.

Levenspiel O. 1980. The Monod Equation: A Revisit and a Generalization to Product Inhibition Situations. Biotechnology and Bioengineering 22: 1671-1687.

Lu Y, Warner R, Sedlak M, Ho N, Mosier NS. 2009. Comparison of glucose/xylose cofermentation of poplar hydrolysates processed by different pretreatment technologies. Biotechnology Progress 25(2):349-356.

Maiorella BL, Blanch HW, Wilke CR. 1984a. Economic evaluation of alternative ethanol fermentation processes. Biotechnology and Bioengineering 26(9):1003-1025.

Maiorella BL, Blanch HW, Wilke CR. 1984b. Feed component inhibition in ethanolic fermentation by Saccharomyces cerevisiae. Biotechnology and Bioengineering 26(10):1155-1166.

Mavituna F, Sinclair CG. 2008. Modelling the Kinetics of Biological Activity in Fermentation Systems. In: McNeil B, Marvey LM, editors. Practical Fermentation Technology. West Sussex, England: John Wiley and Sons, Ltd.

Monod J. 1949. The growth of bacterial cultures. Annual Reviews in Microbiology 3(1):371-394.

Mosier NS, Ladisch MR. 2009. Modern Biotechnology: Connecting Innovations in Microbiology and Biochemistry to Engineering Fundamentals: Wiley.

Olsson L, Hahn-Hagerdal B. 1995. Kinetics of Ethanol Production by Recombinant *Escherichia coli* KO11. Biotechnology and Bioengineering 45: 356-365.

Parawira W, Tekere M. 2011. Biotechnological Strategies to Overcome Inhibitors in Lignocellulose Hydrolysates for Ethanol Production: Review. Critical Reviews in Biotechnology 31(1): 20-31.

Pearl R. 1927. The growth of populations. The Quarterly Review of Biology 2(4):532-548.

Reifenberger E, Boles E, Ciriacy M. 1997. Kinetic Characterization of Individual Hexose Transporters of *Saccharomyces cerevisiae* and Their Relation to the Triggering Mechanisms of Glucose Repression. European Journal of Biochemistry 245:324-333.

For Peer Review

Saloheimo A, Rauta J, Stasyk OV, Sibirny AA, Penttilä M, Ruohonen L. 2007. Xylose Transport Studies with Xylose-Utilizing *Saccharomyces cerevisiae S*trains Expressing Heterologous and Homologous Permeases. Applied Microbiology and Biotechnology 74: 1041-1052.

Taherzadeh MJ, Niklasson C, Lidén G. 1997. Acetic Acid - Friend or Foe in Anaerobic Batch Conversion of Glucose to Ethanol by *Saccharomyces cerevisiae*? Chemical Engineering Science 52: 2653-2659.

Wang DLC, Cooney CL, Demain AL, Dunnill P, Humphrey AE, Lilley MD. 1979. Fermentation and Enzyme Technology. John Wiley & Sons Ltd, Chichester and New York.

Weber C, Farwick A, Benisch F, Brat D, Dietz H, Subtil T, Boles E. 2010. Trends and challenges in the microbial production of lignocellulosic bioalcohol fuels. Applied Microbiology and Biotechnology 87(4):1303-1315.

For Peer Review

Table 1. Estimated model parameters

Parameter	Estimate	Units
μ	0.22	h^{-1}
$v_{max,G}$	1.65	g glucose g^{-1} cells h^{-1}
$v_{max,X}$	0.51	g xylose g^{-1} cells h^{-1}
$Y_{P/G}$	0.35	g ethanol g^{-1} glucose
$Y_{P/X}$	0.41	g ethanol g^{-1} xylose
$Y_{Gly/G}$	0.11	g glycerol g^{-1} glucose
$Y_{Gly/X}$	0.03	g glycerol g^{-1} xylose
$Y_{Xyl/X}$	0.12	g xylitol g^{-1} xylose

Table 2. Indicators of Model Fit

	RSS	R^2
Cell Mass	0.25	0.953
Glucose	10.55	0.993
Xylose	29.09	0.994
Ethanol	4.45	0.998
Glycerol	0.57	0.993
Xylitol	2.99	0.960
Total	*47.89*	

For Peer Review

Table 3. Application of model to alternative initial conditions. The values in the first row represent glucose/xylose/cell mass concentrations (g L^{-1}).

	60/60/4.75		40/40/4.75		60/60/0.60	
	**RSS**	R^2	**RSS**	R^2	**RSS**	R^2
Cell Mass	0.25	0.953	0.59	0.541	0.96	0.979
Glucose	10.55	0.993	44.08	0.924	30.91	0.997
Xylose	29.09	0.994	24.74	0.988	15.08	0.997
Ethanol	4.45	0.998	51.59	0.934	8.00	0.998
Glycerol	0.57	0.993	0.90	0.966	11.18	0.944
Xylitol	2.99	0.960	4.81	0.864	1.49	0.977
Total	*47.89*		*126.70*		*67.62*	

Table 4. Model fit results for a fermentation with the inhibitor acetic acid

	Original Model Parameters		Original Model + AA Inhibition Term		Re-Optimized Model with AA Inhibition Term	
	**RSS**	R^2	**RSS**	R^2	**RSS**	R^2
Cell Mass	0.11	0.969	0.11	0.969	0.11	0.969
Glucose	81.28	0.967	81.28	0.967	81.28	0.967
Xylose	3439.5	0.276	138.29	0.971	138.29	0.971
Ethanol	115.97	0.966	279.28	0.917	15.00	0.996
Glycerol	82.36	-0.254	62.44	0.049	0.16	0.998
Xylitol	234.59	-28.082	111.5	-12.823	0.07	0.991
Total	*3953.81*		*672.90*		*234.91*	

Table 5. Comparison between model parameters for fermentations without and with acetic acid (10 g L^{-1}, pH 5.5)

**Parameter**	**Control No Acetic Acid**	**Acetic Acid 10 g L^{-1}, pH 5.5**
μ	0.22	0.22
$v_{max,G}$	1.65	1.65
$v_{max,X}$	0.51	0.51
$Y_{P/G}$	0.35	0.44
$Y_{P/X}$	0.41	0.44
$Y_{Gly/G}$	0.11	0.06
$Y_{Gly/X}$	0.03	0.06
$Y_{Xyl/X}$	0.12	0.04

For Peer Review

List of Figure Captions

Figure 1. Glucose and xylose consumption by *S. cerevisiae* 424A(LNH-ST). Dashed line designates time when glucose concentration reaches 10 g L^{-1}.

Figure 2. Experimental and model predictions for batch fermentation of glucose and xylose by *S. cerevisiae* 424A(LNH-ST). Experimental values are represented by symbols and predicted values are represented by curves.

For Peer Review

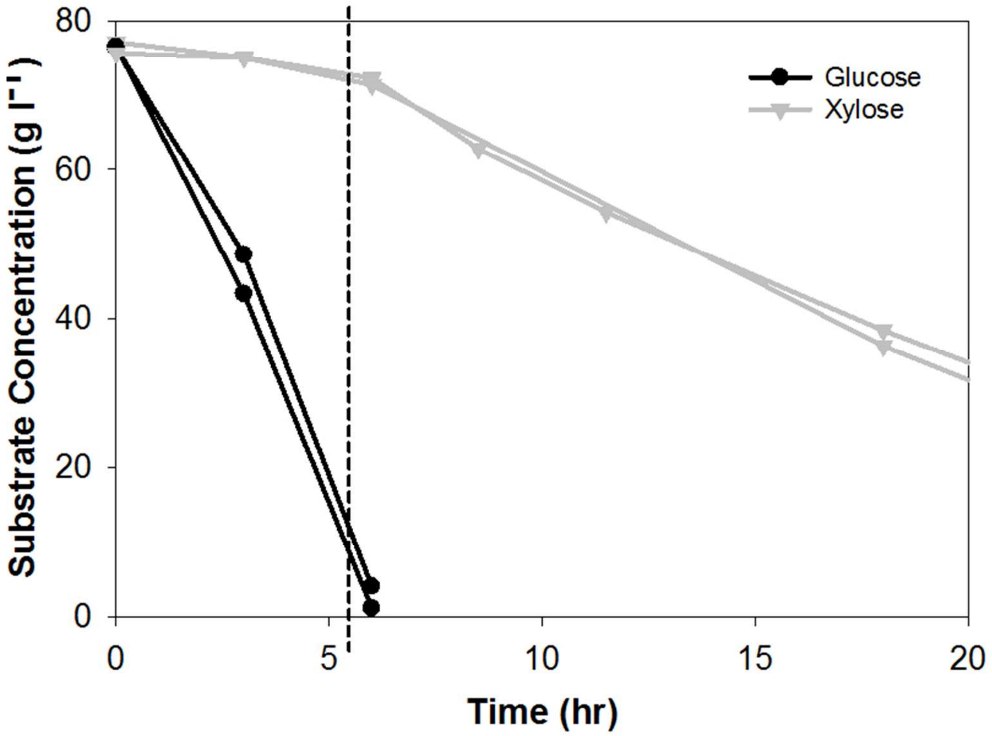

Glucose and xylose consumption by S. cerevisiae 424A(LNH-ST). Dashed line designates time when glucose concentration reaches 10 g L^{-1}.
216x168mm (96 x 96 DPI)

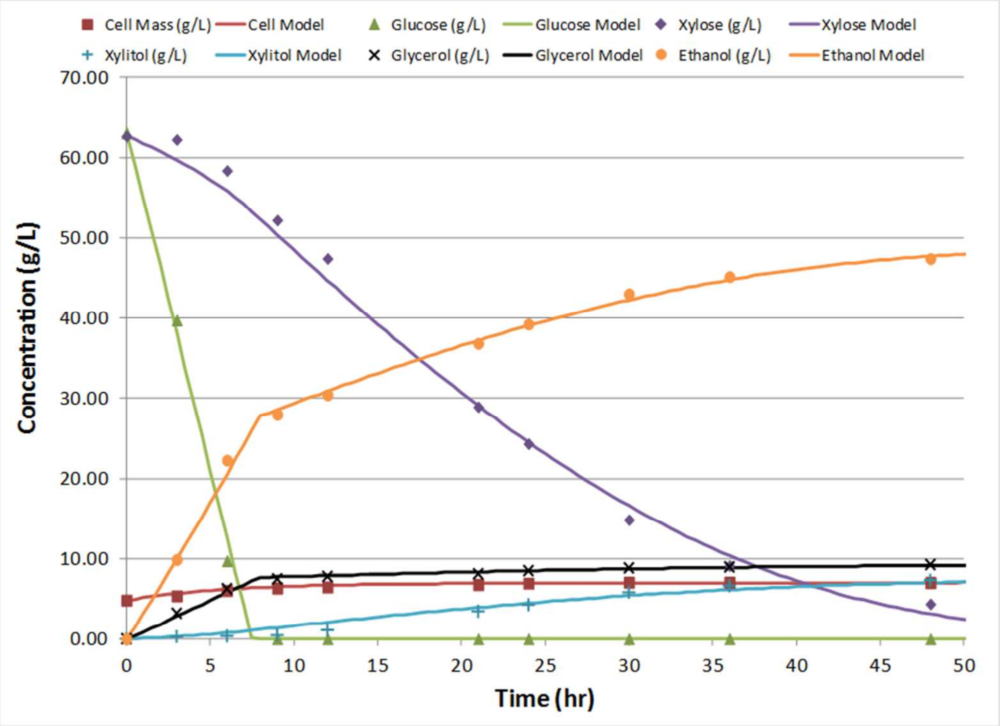

Experimental and model predictions for batch fermentation of glucose and xylose by *S. cerevisiae* 424A(LNH-ST). Experimental values are represented by symbols and predicted values are represented by curves.
227x165mm (96 x 96 DPI)

eview
